# Fabrication and Characterization of Fish Tropocollagen Sponge Enriched with Nanodiamonds for Potential Wound Dressing Applications †

**DOI:** 10.3390/ma19061106

**Published:** 2026-03-12

**Authors:** Bożena Rokita, Dariusz Witkowski, Anna Karczemska, Łukasz Piwowarski, Radosław Wach

**Affiliations:** 1 Institute of Applied Radiation Chemistry, Faculty of Chemistry, Lodz University of Technology, Wróblewskiego 15, 93-590 Łódź, Poland; bozena.rokita@p.lodz.pl (B.R.); radoslaw.wach@p.lodz.pl (R.W.); 2 Division of Medical Apparatus, Institute of Turbomachinery, Lodz University of Technology, Wólczańska 217/221, 93-005 Łódź, Poland; anna.karczemska@p.lodz.pl; 3 Island Clinic, Toruńska 15, 80-747 Gdańsk, Poland; aktivpiwowarski@gmail.com

**Keywords:** fish collagen, tropocollagen, detonation nanodiamonds, resorption, wound dressing, porous scaffold

## Abstract

The development of collagen-based composite materials offers new opportunities for designing bioactive porous structures with tunable properties. This study focuses on sponges or scaffolds fabricated from fish skin-derived tropocollagen combined with detonation nanodiamonds (NDs), aiming to explore how incorporation of NDs and application of radiation, as a potential sterilization method, influence structural and functional characteristics of the material. Freeze-dry methods of sponge fabrication resulted in a bilayered structure of open porosity, with microporosity at the top and a microchannel at the lower part of the material. The sponges demonstrated mechanical properties with relatively low elongation of below 10%, while the maximum stress was reduced by ca. 20% due to irradiation. Hydration and absorption experiments, mimicking the resorption of collagen in physiological conditions of expected application as wound dressing material, demonstrated controllable fluid uptake and gradual material dissolution, taking place over several hours, depending essentially on the irradiation treatment and morphological characteristics of the sponge. These findings highlight the versatility of collagen–nanodiamond composites as platforms, in which structural design and processing parameters control performance. Moreover, they provide a strong indication of the expected behavior of collagen–nanoparticle systems, including those incorporating NDs modified to impart specific biological functionality, such as antimicrobial activity.

## 1. Introduction

Collagen is the most abundant structural protein in vertebrate extracellular matrices and plays a central role in conferring tissue integrity, mechanical strength, and biological function. All collagens share the characteristic (Gly–X–Y)n motif, where X and Y are most frequently proline and hydroxyproline, which stabilize the triple-helical conformation through hydrogen bonding. Nevertheless, collagens exhibit significant diversity in amino acid composition, supramolecular arrangement, and tissue distribution. With over 25 known types and accounting for ca. 30% of mammalian proteins, collagen versatility relies on sequence and organizational diversity [1]. The tropocollagen protein, which comprises three polypeptide α-chains, is the foundational unit that assembles into fibrils and fibers, imparting multiscale mechanical performance through hierarchical organization [2]. The functional extent of collagen arises from differences in amino acid composition, helix length, helix packing, and supramolecular architecture, which collectively modulate mechanical behavior, degradation kinetics, and bioactivity in material contexts [3,4]. Its widespread use in biomaterials is due to its intrinsic biocompatibility, bioactivity, biodegradability, and low antigenicity, supporting applications across wound dressings, scaffolds, and regenerative material systems [3,5,6].

Growing emphasis on sustainable sourcing and bioeconomy has driven interest in fish-derived collagen, particularly from processing by-products (skin, bones, and fins) [7,8,9]. Compared with mammalian collagen, fish-derived materials offer reduced zoonotic risk and fewer cultural/ethical constraints, efficient extraction from waste streams, aligning with circular manufacturing, and distinct thermal behavior as well as composition, enabling alternative processing routes [10,11,12,13].

Marine collagens typically exhibit lower proline and hydroxyproline content, translating to a reduced denaturation temperature (~25–30 °C) versus mammalian collagen (~39–40 °C) [14,15,16]. While lower thermal stability constrains high-temperature fabrication and certain load-bearing uses, it also facilitates mild processing, retaining bioactive motifs and cell-instructive cues, critical for integration in tissue engineering and regenerative materials, and enables hydrated/porous architectures that closely mimic the extracellular matrix. Fish-derived collagen offers high water-binding capacity, which is advantageous for moisture management in wound dressings and cosmetic formulations [17,18]. Specifically, it provides a sustainable, bioactive, and process-friendly alternative for fabricating porous sponges, films, hydrogels, and nanofibrous meshes that can be adapted to regulate drug release, cell attachment, and degradation profiles [19]. It also demonstrates cytocompatibility and supports cell adhesion and proliferation (e.g., fibroblasts), while its porosity, swelling behavior, and degradation rate can be tuned through crosslinking and blending strategies [20]. Moreover, in order to address some limitations and tailor performance, hybrid collagen-based systems may integrate synthetic polymers, inorganic phases, and bioactive agents, with the following being examples: (a) poly(ε-caprolactone) (PCL) and hyaluronic acid hybrids for the enhancement of physicochemical properties and enabling controlled release, expanding utility in scaffolding and advanced dressings [21]; (b) marine-derived silica (sponges) combined with collagen yields bioactive, 3D porous scaffolds with promising osteoconductive potential for bone tissue engineering [22]; (c) an electrospun mesh of PCL and tilapia-derived collagen with antibiotics demonstrate active infection control and tissue repair, particularly in complex wounds [23]; and (d) natural bioactives (e.g., chitosan nanoparticles, plant extracts) introduce antimicrobial and pro-regenerative functions, enabling multimodal dressings and scaffolds [24].

Special attention is paid to inorganic nanomaterial composites encompassing montmorillonite, halloysite nanotubes, titanium dioxide, silica nanoparticles, zinc oxide, and carbon-based nanomaterials. They can be incorporated into a synthetic or natural polymeric matrix, or even protein-based platforms to render them biologically functional, especially in terms of hemostatic or antimicrobial activity [25,26]. Graphene derivatives, carbon dots, carbon nanotubes, carbon nanofibers, and nanodiamonds are well-known examples of carbon nanomaterials that have been employed in biomedical research due to their unique physicochemical properties, biocompatibility, and multifunctionality. Among them, nanodiamonds (NDs) stand out as a distinctive class of carbon nanoparticles, characterized by a crystalline diamond core of nanoscale dimensions (2–10 nm) and a surface rich in oxygen-containing functional groups [27]. These features confer exceptional chemical stability, high surface areas, and tunable surface chemistry, enabling applications across drug delivery, tissue engineering, and wound healing. Numerous in vitro and in vivo studies confirm the low cytotoxicity of NDs and their favorable interactions with cells [28,29].

The growing prevalence of multidrug-resistant bacteria further underscores the need for innovative antimicrobial strategies. Surface-modified NDs exhibit antibacterial and anti-biofilm properties [30,31]. In wound care, modified NDs have also demonstrated antibacterial activity, the promotion of cell adhesion, the modulation of inflammatory responses, and potential as carriers for bioactive molecules [32,33]. Their incorporation into biopolymeric matrices may enhance the structural stability and biological performance of wound dressings while not altering the high porosity and moisture retention of the carrier-critical parameters for effective wound management. Nanodiamonds, owing to their ability to enhance hydrophilicity, degradation behavior, and cell adhesion in polymer-based composites, are increasingly recognized as promising functional additives for the development of advanced tissue engineering scaffolds and neural regeneration [34,35,36].

The combination of a biologically active, extracellular matrix-comprising collagen with a multifunctional nanomaterial may offer a synergistic effect in enhancing the functional properties of protein-based dressings. In this context, the present work explores a novel composite based on the integration of fish-derived tropocollagen with nanodiamonds in the form of porous sponges, manufactured by freeze-drying (lyophilization) intended for potential wound dressing applications. Previous work of autors [37], as a brief two-page technical announcement, introduced the concept but did not provide fabrication details or quantitative characterization; the present article substantially explores the subject. The elimination of water through sublimation, a well-known technique, enables the formation of highly porous, lightweight scaffolds, typically with interconnected pore networks, facilitating exudate absorption, oxygen diffusion, yet protecting the wound from external contamination, and providing a natural microenvironment for tissue regeneration—features essential for optimal wound healing. The incorporation of nanodiamonds into the tropocollagen matrix is hypothesized not to disrupt the mechanical integrity of the collagen structure, and may introduce additional bioactive function, such as antimicrobial effects and the modulation of cell–material interactions—modified NDs are reported to act that way [32]. Based on the literature surveyed, the fabrication and initial characterization of freeze-dried sponges composed of fish-derived tropocollagen with detonation nanodiamonds appear to be unexplored, indicating a promising biomaterial platform for wound dressing and tissue engineering applications.

A major challenge in the application of protein-based solid biomaterials lies in their sterilization. Devices composed of biomolecules, particularly those in which conformational changes result in the loss of biological activity, cannot be sterilized using thermal or chemical methods [38]. Consequently, radiation-based sterilization represents the only viable approach for solid collagen materials such as a sponge, fibers, or film. Therefore, the aim of this study was to investigate the behavior of the nanocomposite with respect not only to the incorporation of nanodiamonds (NDs) within the collagen matrix, but primarily to the effects of radiation sterilization. Particular attention was given to irradiation-induced alterations in the hydration properties of the collagen sponge and its resorption under wound-mimicking conditions.

## 2. Materials and Methods

### 2.1. Materials

The collagen was extracted from silver carp skin (Sancoll Co. Ltd., Rzeszów, Poland). It was delivered chilled in the form of a homogeneous, viscous gel with a pH of ca. 4.2, greyish transparent, and free of visible impurities. The concentration of tropocollagen within the gel was ca. 2.8%, and it contained lactic acid as a preservative. Detonation nanodiamonds (NDs) were purchased from Ray Techniques Ltd., Jerusalem, Israel, NDs were heat treated at ca. 450 °C for 5 h in air in order to introduce oxygen containing groups at the surface, such as hydroxyl, ketone, aldehyde, and also carboxylates, as this modification was proved to render ND antibacterial activity [30].

### 2.2. Collagen Sponge Preparation

To fabricate collagen-based sponges, a freeze-drying (lyophilisation) technique was employed. Nanodiamonds were introduced into the collagen by mixing of the gels with an ND–water suspension in a 7:3 ratio. In order to prepare the ND suspension, NDs in a concentration of 0.5% were dispersed in water with an ultrasonic homogenizer (Omni Sonic Ruptor 400 sonicator, Omni International, Kennesaw, GA, USA), and further diluted. The ultrasonic treatment for ca. 1 h resulted in the rupture of ND agglomerates (below < 100 nm average diameter) intrinsically formed during storage. The collagen gel with NDs were distributed into trays and freeze-dried, that is, frozen at −75 °C for at least 24 h and subjected to sublimation in a lyophilizer (Labconco Corporation, Kansas City, MO, USA) under ca. 0.10 mPa for 48 h. The concentration of nanodiamonds in the final lyophilized product was approximately 1 wt%. Such concentration of nanodiamonds was deliberate, as it has been used in nanodiamond–polymer research because it easily maintains the balance between stability of the matrix and good particle dispersion, avoiding agglomeration, and providing biological safety [36]. Moreover, the present work aims to demonstrate the feasibility of producing a collagen–nanodiamond composite material, where 1 wt% NDs serve as a reasonable reference concentration commonly used for analogous proof-of-concept studies.

### 2.3. Collagen Sponge Irradiation (Sterilization)

The lyophilized sponges were cut into strips of 10 mm width to obtain the final shape required for subsequent testing. The samples were grouped, sealed in PE foil, and subjected to irradiation at doses of 25 kGy and 40 kGy, in order to simulate minimum and maximum dose of typical distribution during industrial sterilization. The irradiation of collagen sponges was accomplished with an electron beam (EB) from a linear accelerator (ELU-6, Elektronika, Moscow, Russia) generating a beam of electrons of 6 MeV energy. The dose rate was ca. 1 kGy/min, and alanine dosimetry was used. The processing did not increase the temperature of the material by more than 5 °C.

The collagen sponge samples were without and with NDs; their description is presented in Table 1.

### 2.4. Morphological Analysis of Collagen Sponges

The macroscopic photographs of the collagen sponges were taken with a Nikon digital camera. The morphology or the internal microporosity of the sponges was examined using a scanning electron microscope (Hitachi TM-1000, High-Technologies Corporation, Tokyo, Japan). The samples were coated with gold prior to observation. Pore diameter measurements were performed using the Feret diameter. Feret diameter is the distance between two parallel lines tangent to an object’s outline, measured in a specified direction; in practice, this makes it possible to characterize the size and shape of irregular structures such as pores [39]. To quantify the transversal pore geometry in the microchannel section, image analysis was performed with a minimum size threshold of 0.150 mm to focus on the macroporous features, typically relevant for cell infiltration and nutrient transport, but with the view of an anticipated application essential for the absorption of wound excretion and collagen dissolution.

### 2.5. Static Tensile Tests

Collagen sponge samples were subjected to tensile strength testing using a Zwick Roell Z 2.5 universal testing machine (Zwick Roell, Ulm, Germany) equipped with a 100 N load sensor. A collagen sponge approximately 10 mm thick, naturally consisting of two different microporous sections on its thickness, was cut on its shorter side into strips of approximately 70 mm and 10 mm in width. The dimensions of every sample were measured prior to the experiment. The specimens were mounted in fixed grips specifically designed for fragile materials, with a 30 mm gap, and the measurements were carried out with an extension speed of 10 mm/min, under room temperature and ca. 40–45% humidity. At least five specimens of every material were tested, based on which the maximum tensile stress (equal to stress at break) and elongation at break were determined.

### 2.6. Resorption Study

The aim of this experiment was to evaluate an impact of irradiation on the dissolution of the collagen sponge samples under controlled conditions mimicking a wound environment. Collagen sponges in the form of cubes of ca. 10 × 10 × 10 mm^3^ dimensions were placed on a wound-mimicking sterile hydrogel prepared by radiation-induced synthesis, composed of <10%wt polyvinylpyrrolidone with minor additives of polyethylene glycol and agar [40]. The hydrogel of ca. 10 mm thickness allowed for the realistic simulation of a a wound, as it was pre-swollen in PBS buffer (Gibco), and the pH was adjusted to 4.5, corresponding to conditions in a wound.

Experiments were conducted at 37 °C on a heating plate with the collagen sponge placed directly on the hydrogel. The study was performed in two conditions: under a cover, simulating a dressed wound, and uncovered without any additional top dressing. At defined time intervals, photographic documentation was carried out using a Nikon digital camera (Nikon Corporation, Tokyo, Japan). Subsequently, the height of the collagen sponges in photographs was measured using ImageJ software (version 1.54p, National Institutes of Health, Bethesda, MD, USA) to assess the degree of collagen absorption or dissolution that simulates the collagen resorption rate in a realistic environment.

## 3. Results and Discussion

The lyophilized collagen samples containing nanodiamonds were characterized using scanning electron microscopy (SEM) to assess their microstructural features; a static tensile test was conducted to evaluate mechanical properties, and solubility tests were carried out in order to evaluate the dissolution of collagen sponge in simulated wound conditions.

While numerous collagen-based lyophilized sponges incorporating various additives (e.g., hyaluronic acid, alginate, chitosan, curdlan, and antibiotics) have been reported [41,42,43,44,45,46], no prior publications were identified that describe freeze-dried sponges prepared from fish-derived tropocollagen combined with detonation nanodiamonds. This study therefore provides a proof of concept for this specific composite system. Numerous authors have highlighted the application of collagen-based sponges in wound healing, and the review paper by Park et al. indicates that nanodiamonds also exhibit favorable properties for promoting tissue repair [33]; therefore, a novel material combining fish-derived tropocollagen and nanodiamonds may potentially serve as an effective wound dressing.

### 3.1. Collagen Sponge Morphology

**Figure 1:**
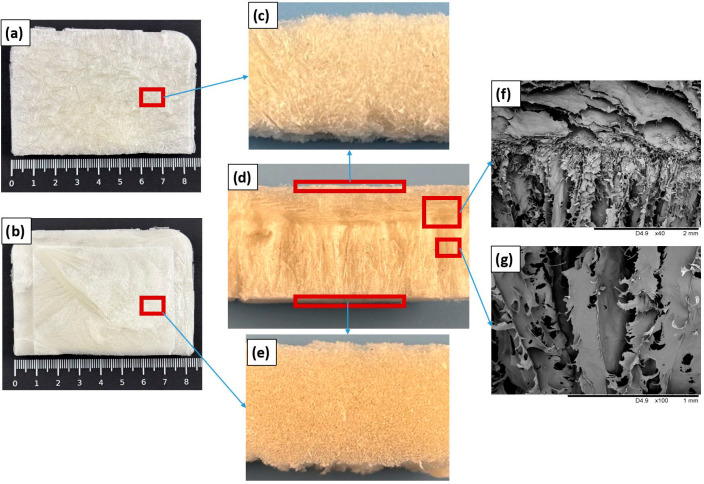
Collagen sponge showing different regions and structural features: (**a**) top surface (microsponge side, macroscopic view); (**b**) bottom surface (microchannel side, macroscopic view); (**c**–**e**) magnified views acquired with a digital camera—height and width of the depicted samples were 10 mm: (**c**) top view (microsponge side); (**d**) lateral cross-section; (**e**) bottom view (microchannel side); and (**f**,**g**) SEM images: (**f**) interface between top and lateral microchannels, (**g**) transverse cross-section showing internal microchannels.

To illustrate the overall structural organization of the collagen sponges, Figure 1 presents images of different regions and orientations of the material. Macroscopic views capture the top (a), designated as ‘microsponge’, and bottom (b), designated as ‘microchannels’, surfaces, while magnified digital camera images show detailed (c) top, (d) lateral cross-section, and (e) bottom perspectives. SEM images further highlight the microstructural features, including (f) the interface between top microsponge and lateral microchannels and (g) a transverse cross-section revealing internal microchannels. This microstructure results from the manufacturing method, that is, liophilization of collagen gel. A large top surface facilitates quick sublimation, producing non-oriented interconnected porosity, whereas sublimation of water from a deeper part of the frozen material generates microchannels. These may result in different behaviors of the sponge or scaffold depending on the application side, for instance, covering wounds in anticipated application—this will be developed later in the article.

**Figure 2:**
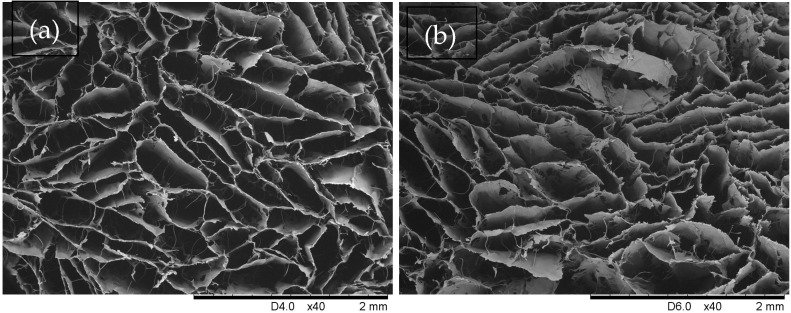
SEM images of exemplary collagen sponge, cross-section through the thickness of the dressing (microchannel side) used for pore evaluation: (**a**) without NDs, (**b**) with NDs.

The analysis of the microstructural architecture of the scaffolds revealed a highly porous, interconnected network with irregular cellular morphology—SEM images in Figure 2a,b. The quantitative results, presented in Table 2, indicate that the samples possess a broad pore size distribution. The standard deviation (SD) values are substantial (ranging from ±0.158 mm to ±0.328 mm), which is consistent with the anisotropic and heterogeneous nature of the pore structure observed in the micrographs.

The global mean pore size (Feret diameter) was calculated to be 0.292 mm, and it appeared to be independent of the absence or presence of NDs. The large standard deviations relative to the mean values—exemplified by sample (Figure 2b) (0.310 ± 0.328 mm)—highlight the wide variance in pore dimensions. This heterogeneity is often advantageous for tissue engineering applications, as the smaller pores provide a large surface area for cell attachment, while the larger ones facilitate efficient bulk fluid transport and vascularization deep within the scaffold. Several studies have emphasized the critical role of pore geometry—including size, connectivity, and whether pores form open-channel networks or are partially closed—in determining the functional properties of collagen-based sponges. These works have demonstrated the feasibility of producing collagen scaffolds with high porosity and diverse pore architectures, highlighting the potential for the fabrication of sponges with specific geometrical characteristics [47,48,49]. Moreover, studies indicate that a broad pore size distribution and controlled structural heterogeneity in freeze-dried collagen scaffolds may positively influence wound healing outcomes. Scaffolds containing both small pores (typically < 100 µm) and larger interconnected pores (>150–250 µm) provide a dual functional advantage: smaller pores enhance capillary-driven fluid absorption and moisture retention, while larger pores facilitate fibroblast migration, neovascularization, and oxygen diffusion. Such hierarchical porosity more closely mimics the extracellular matrix architecture and supports different stages of wound healing, from exudate management to tissue remodeling. Therefore, the large variation in pore size obtained in the current study may be beneficial for the anticipated application. However, the beneficial effect of pore heterogeneity depends critically on maintaining sufficient interconnectivity and mechanical integrity, as excessive irregularity may compromise scaffold stability and lead to uneven degradation [50]. Therefore, controlled multiscale porosity rather than random heterogeneity appears to be optimal for advanced collagen-based wound dressings. In the collagen sponge specimens, the porosity estimated from the mass of solid collagen, with the assumption that the dimensions of the samples did not change upon freeze-drying (confirmed by the measuring and weighting of exemplary specimens), was in the range of 97–98%. This high porosity is indicative of an open-cell structure favorable for cellular infiltration/fluid permeability if applicable as an internal scaffold.

### 3.2. Tensile Strength of Collagen Sponge

The tensile stress and elongation at break determined for each material are presented, together with standard deviations for both, in Table 3. The maximum stress ranged from 39.8 kPa to 53.3 Pa, whereas the average maximum stress for all samples was 45.8 kPa. The average elongation was relatively small, 6.8%, extending from 6.1 to 8.3%, indicating materials with limited elasticity. The presence of NDs in the sponge matrix has no apparent effect on mechanical strength and elongation of materials. The key factor influencing the mechanical properties of collagen sponges is the effect of irradiation—radiation is supposed to be used for product sterilization. Comparing the values averaged for each dose, it is evident that irradiation with 25 kGy did not change elongation but decreased the maximum strain by ca. 20%; note that extension of irradiation up to 40 kGy does not influence the strength of the collagen. Disruption of the three-dimensional structure of tropocollagen, that is, breakage of hydrogel bonds, observed in collagen irradiated in a hydrated state may not be the principal factor responsible for those changes, as the current processing was carried out in a solid state. In a hydrated state, crosslinking of collagen was observed, but contrarily, the collagen materials irradiated in a solid state undergo scission of polypeptide mainchain bonds; therefore, it is more likely that this is the reason for the reduction in mechanical performance [51]. These results provide insight into the mechanical behavior of the freeze-dried sponge or a scaffold collagen material, thus supporting the further evaluation of their suitability for biomedical applications, particularly in wound dressing design [33].

### 3.3. Collagen Sponge Hydration Under Simulated Wound Conditions

The liquid absorption of the collagen sponges was evaluated under conditions simulating a moist wound environment. The samples were placed on a pre-swollen, wound-mimicking hydrogel and incubated at physiological temperature (37 °C). Changes in sponge geometry over time were monitored as an indirect measure of liquid uptake and structural integrity. Initially, the experiments were conducted under a cover, simulating a dressed wound, to assess the influence of a dressing-like barrier on the absorption process.

**Figure 3:**
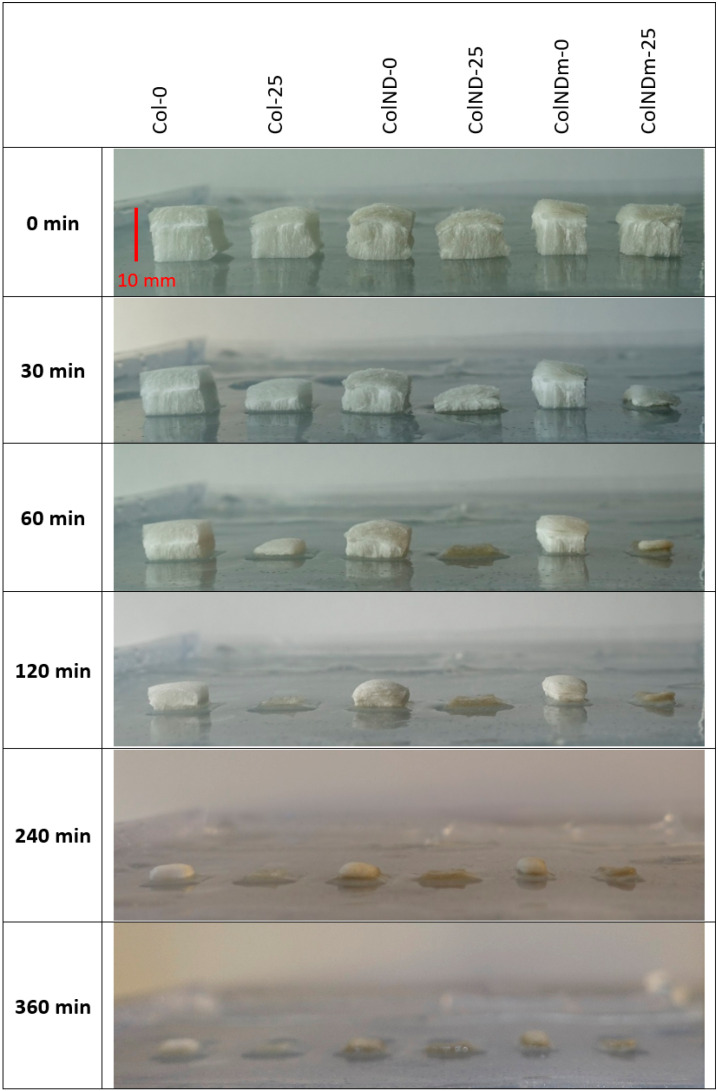
Collagen absorption experiment under a cover at 37 °C—images of collagen sponges with an initial height of ca. 10 mm on a hydrogel substrate.

The images in Figure 3 indicate that irradiation influences early-stage absorption. The presence of nanodiamonds, either original or thermally treated, does not substantially affect the absorption kinetics. Nevertheless, irradiated sponges (25 kGy; columns 2, 4, and 6) exhibit a visibly faster reduction in height compared to non-irradiated samples (0 kGy; columns 1, 3, and 5), whereas all samples appear fully wetted after 360 min. These observations provide a qualitative comparison of the hydration process under covered conditions.

**Figure 4:**
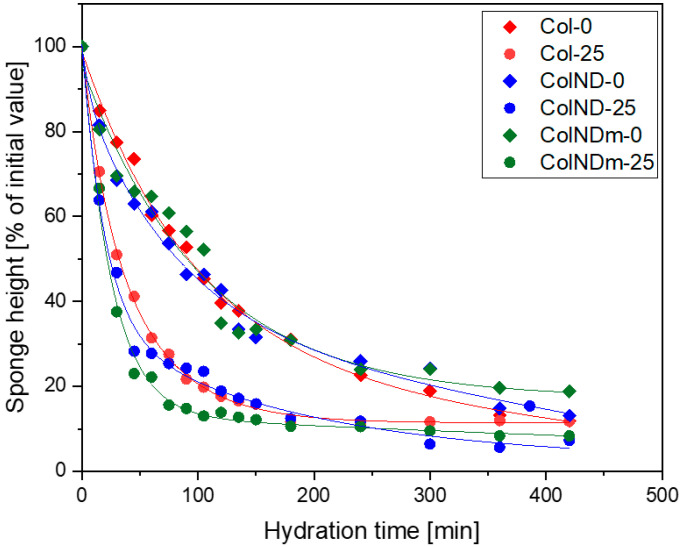
Height changes of collagen sponges during hydration under a cover, simulating a dressed wound, for all individual samples—Col, ColND, and ColNDm (0 and 25 kGy).

Quantitative analysis of collagen resorption (Figure 4) was translated into a half-time of hydration (or reduction in sample height). This confirms that the presence of NDs does not influence the absorption of collagen sponges, but irradiation accelerates its absorption. Irradiated sponges reach approximately 50% hydration within 30 min, whereas non-irradiated sponges reach the same level after about 100 min—comparison in Table 4. These results indicate that irradiation primarily influences the rate of sponge hydration under occlusive conditions, while the final hydration extent is similar for all types of samples.

**Figure 5:**
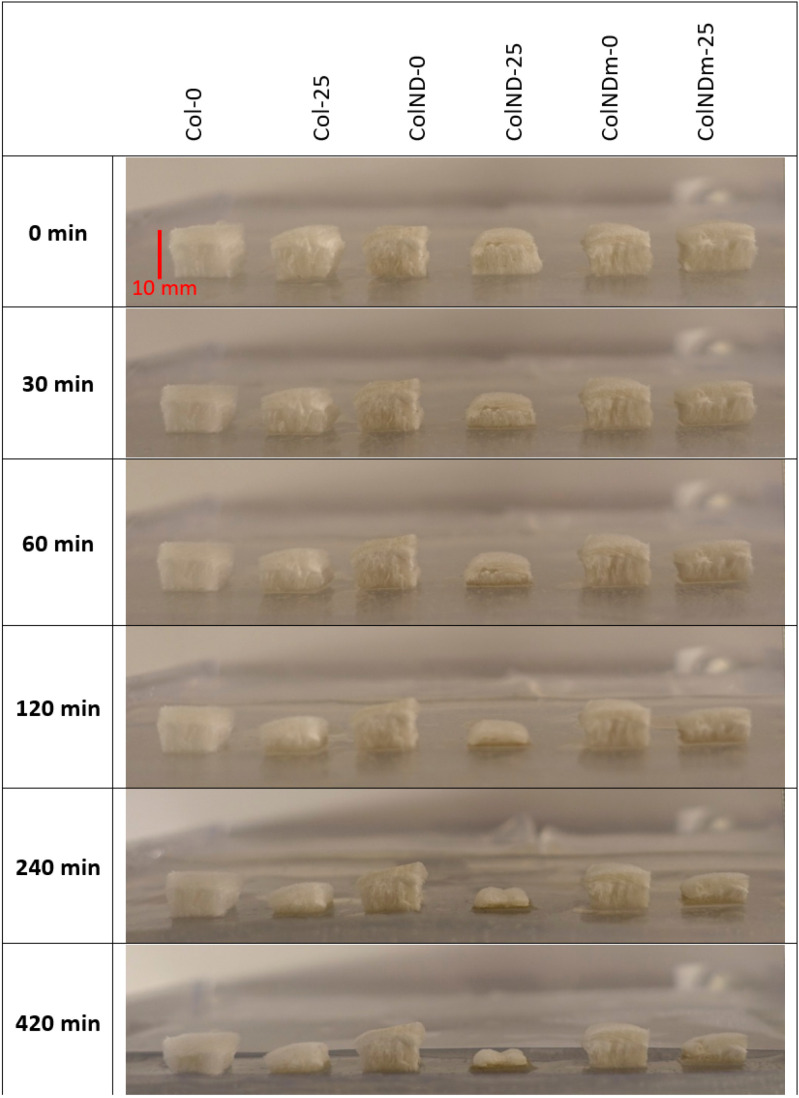
Collagen absorption experiment without a cover at 37 °C—images of collagen sponges with an initial height of ca. 10 mm on a hydrogel substrate.

To eliminate the influence of occlusion on the absorption behavior of collagen sponges, experiments were performed without a cover, simulating an undressed wound. Samples were placed directly on the pre-swollen wound-mimicking hydrogel at 37 °C, with natural access to surrounding air, and changes in sponge geometry were monitored over a time range of 0–420 min. Figure 5 presents representative images showing macroscopic hydration and structural changes under these uncovered conditions.

Compared to the covered system, the hydration of uncovered sponges was slower, reflecting the absence of an occlusive barrier. Irradiated sponges (25 kGy) exhibited faster reduction in height and partial collapse of their original three-dimensional structure than non-irradiated samples, although the differences were less pronounced than under covered conditions. Even after 420 min, all sponges were only partially wetted, yet the presence of nanodiamonds (original NDs or thermally treated NDs) did not visibly alter the hydration pattern.

**Figure 6:**
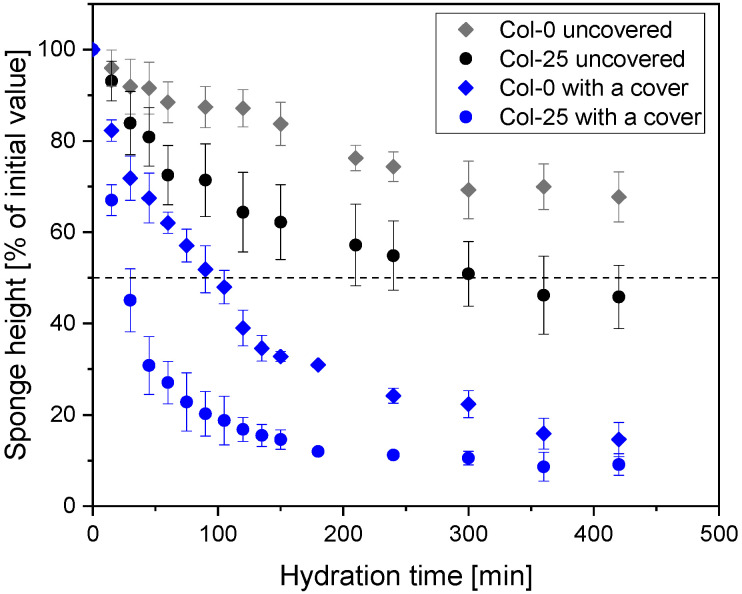
Comparison of averaged height changes of collagen sponges with and without a cover, comparing irradiated (25 kGy) and non-irradiated samples.

To complement the qualitative observations, the averaged changes in height for all sponges were quantified under both covered and uncovered conditions. Consecutive data will be presented as averaged separately for non-irradiated and irradiated samples, as the impact of the NDs’ presence is minor. Figure 6 compares irradiated (25 kGy) and non-irradiated (0 kGy) samples. Quantitative analysis confirms that occlusion strongly accelerates hydration: sponges under a cover show a substantially faster decrease in height than uncovered samples. Within both environmental conditions, irradiation consistently promoted faster height reduction, with 25 kGy samples losing height more rapidly than non-irradiated ones. These results indicate that while irradiation primarily affects early-stage absorption, occlusion is the dominant factor determining overall hydration kinetics, and full resorption of future collagen dressing will be achieved faster if it is covered to maintain a wet environment.

**Figure 7:**
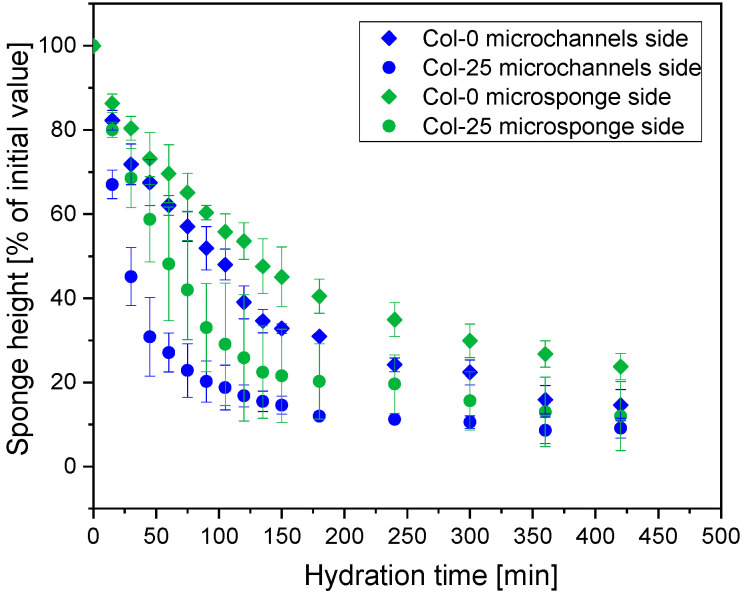
Comparison of averaged height changes of non-irradiated and irradiated collagen sponges with microchannel and microsponge sides placed down on a hydrogel substrate, under a cover.

Figure 7 shows the influence of sponge orientation on the hydrogel under a cover, comparing the standard orientation (microchannels facing down, as in the preceding experiments) and the reversed orientation (microsponge facing down) for both non-irradiated (0 kGy) and irradiated (25 kGy) samples. Quantitative analysis of height changes indicates that orientation affects the rate of hydration. Sponges in the standard orientation (microchannels in contact with the hydrogel; blue series) exhibited faster hydration compared to the reversed orientation (microsponge in contact; green series). The effect was particularly pronounced in non-irradiated samples. This apparently results from the structural anisotropy within the collagen sponges that modulates liquid absorption, as it depends on nature of the sponge microstructure at the interface with the hydrogel—compare Figure 1.

Consistent with the overall experimental observations, irradiation appears to accelerate early-stage hydration. Samples exposed to 25 kGy lost height more rapidly than non-irradiated counterparts, regardless of orientation. These findings indicate that both sponge structure (i.e., the surface facing the wound) and the irradiation, or radiation sterilization, influence absorption kinetics. Occlusion remains the dominant factor controlling overall hydration, as demonstrated in the previous experiments.

Overall, the combined results from all experiments demonstrated that (i) irradiation primarily affects the rate of early absorption, (ii) occlusive conditions substantially accelerate hydration, and (iii) the internal architecture of the sponge, including surface orientation, can modulate liquid uptake. The presence of nanodiamonds (original NDs or thermally treated NDs) did not significantly alter these trends, indicating that their effect on absorption kinetics is negligible under the tested conditions.

**Figure 8:**
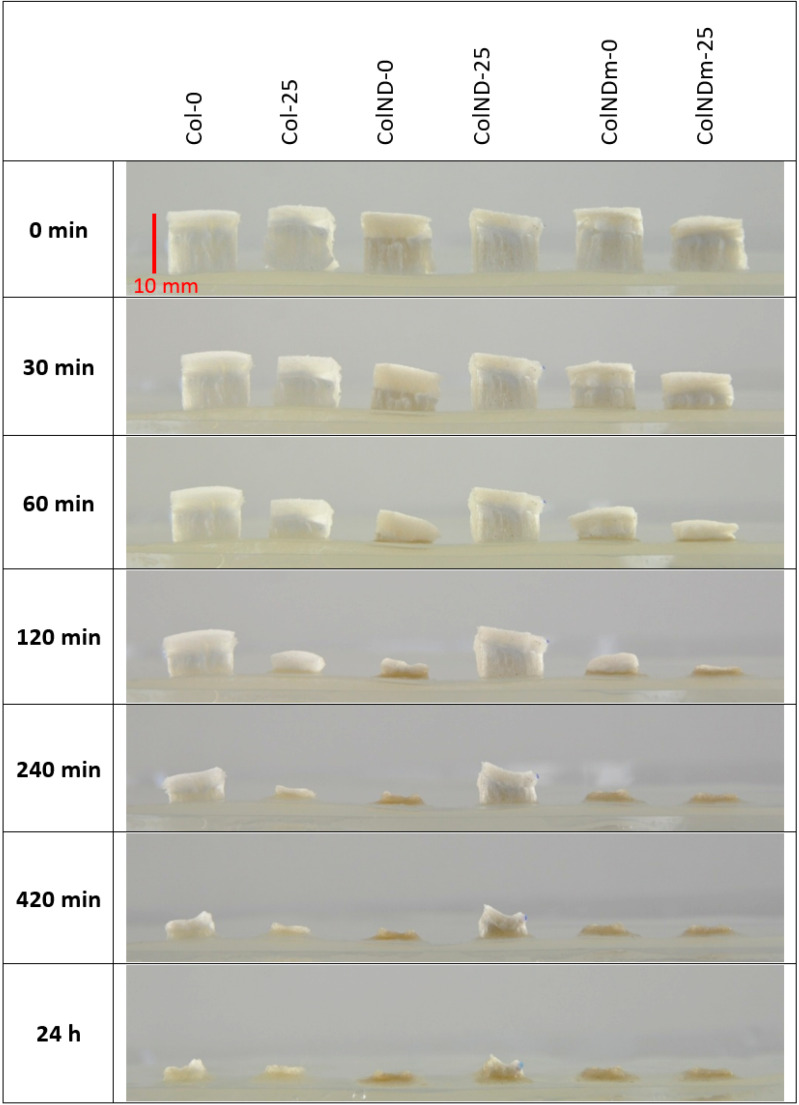
Collagen absorption experiment under a cover at 37 °C—images of collagen sponges (non-irradiated and irradiated at 25 and 40 kGy) with an initial height of ca. 10 mm on a hydrogel substrate.

To further assess the effect of higher irradiation doses and prolonged hydration under occlusive conditions, the hydration profile of collagen sponges irradiated at 0, 25, and 40 kGy were qualitatively compared using macroscopic imaging (Figure 8) and quantitatively determined with respect to kinetics and half-time of resorption (Figure 8, Table 4).

Figure 8 presents macroscopic images illustrating the hydration and long-term structural evolution of collagen sponges exposed to irradiation under covered conditions at 37 °C. As hydration progressed, all samples exhibited a gradual reduction in volume and loss of their original three-dimensional structure. However, differences between non-irradiated and irradiated sponges are apparent—collagen exposed to irradiation (25 and 40 kGy; columns 2, 3, 5, and 6) showed a noticeably faster collapse compared to non-irradiated controls (columns 1 and 4), with pronounced flattening after 120–240 min. Although some qualitative differences between the 25 and 40 kGy doses could be observed at earlier time points, both irradiated specimens ultimately underwent extensive structural degradation over a comparable time scale. After prolonged hydration (up to 24 h), continued deterioration was evident for all samples, including non-irradiated sponges that lost substantial structural integrity, whereas irradiated samples upon hydration reduced their dimensions and eventually turned into a thin residue on the hydrogel surface. Consistent with earlier observations, the presence of nanodiamonds (original NDs) did not macroscopically alter the degradation behavior at any irradiation dose.

**Figure 9:**
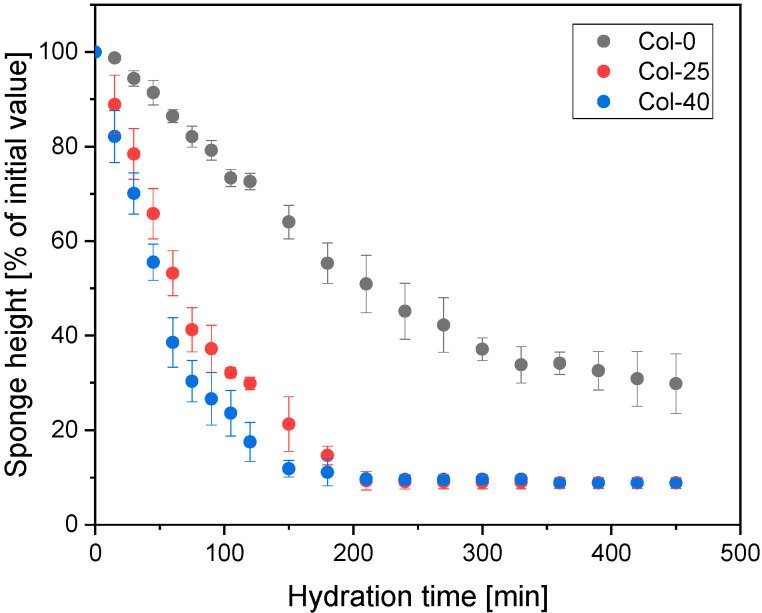
Comparison of averaged height changes of non-irradiated and irradiated groups at 25 and 40 kGy collagen sponges on a hydrogel substrate, under a cover.

The quantitative results presented in Figure 9 confirm the macroscopic observations from an analysis of pictures in Figure 8 and enable a more detailed comparison of hydration kinetics as a function of irradiation dose. Non-irradiated samples exhibited the slowest rate of height loss, retaining approximately 30% of their initial height after 350 min. In contrast, irradiation significantly accelerated sponge hydration and collapse. A comparison between the two irradiation doses reveals a dose-dependent effect during the early stages of hydration: sponges irradiated with 40 kGy showed a slightly faster initial decrease in height than those irradiated with 25 kGy. For example, after 50 min of hydration, the 40 kGy samples retained approximately 55% of their initial height, whereas the 25 kGy samples retained about 65%. At longer hydration times (beyond approximately 200 min), the height loss kinetics of both irradiated groups converged, reaching a similar plateau at around 10% of the initial height—the hydrated residue of collapsed collagen material. Although there was no difference in the mechanical properties between samples irradiated with two doses, this experiment demonstrated that increasing the irradiation dose above 25 kGy influences early hydration kinetics. The absolute hydrated state under occlusive conditions remains comparable.

Table 4 summarizes the time required for collagen sponges to reach 50% hydration (t_1/2_) under different experimental conditions. Under covered conditions, sponge orientation (microchannel vs. microsponge sides facing down) has an evident influence on hydration kinetics. Non-irradiated sponges (0 kGy) with the microchannel side placed on the hydrogel substrate reached 50% hydration faster (t_1/2_ ca. 90 min) than those placed with the microsponge side (t_1/2_ ca. 128 min). A similar orientation-dependent trend was observed for irradiated samples (25 kGy), with the microchannel side exhibiting faster hydration (t_1/2_ ca. 24 min) compared to the microsponge side (t_1/2_ ca. 54 min).

Irradiation markedly accelerated the hydration process regardless of sponge orientation. For covered samples placed on the microchannel side, irradiation reduced t_1/2_ from ca. 89 min to ca. 24 min, while for the microsponge side the corresponding reduction was from ca. 128 min to ca. 54 min. These results confirm that irradiation primarily affects the kinetics of fluid uptake, whereas the relative differences between sponge orientations are preserved.

In the uncovered condition, hydration proceeded substantially slower. Non-irradiated sponges (0 kGy) did not reach 50% hydration within the 450 min experimental time frame, whereas irradiated sponges (25 kGy) reached t_1/2_ at ca. 300 min. This finding highlights the combined influence of irradiation and occlusion on hydration kinetics, indicating that both factors promote rapid fluid uptake.

Overall, the results demonstrate that the hydration and dissolution properties of collagen sponges can be modulated by irradiation dose, sponge orientation, and the presence of occlusion (covering). Structural features related to sponge architecture, such as pore orientation and surface morphology, influence fluid penetration pathways, while irradiation alters the collagen structure due to the scission of the polypeptide backbone, leading to accelerated hydration. The experimental configuration, particularly whether the sponge is applied under occlusive conditions, further controls the rate of fluid uptake. Together, these parameters provide a basis for tailoring the hydration kinetics of collagen-based wound dressings to specific application requirements.

Although collagen-based dressings are well recognized for their role in exudate absorption and maintaining a moist wound microenvironment, there is a lack of studies systematically quantifying the hydration kinetics of three-dimensional pure collagen sponges under conditions closely mimicking a wound—including occluded versus non-occluded scenarios, contact with wound-like substrates, and controlled pore orientation—which limits direct quantitative comparisons with the literature [52,53,54]. Previous reports on collagen irradiation suggest that it can alter the porosity, mechanical properties, and/or swelling behavior of collagen materials, indirectly supporting our observations of accelerated early absorption at 25–40 kGy; however, previous studies were conducted under conditions differing from the current experiment (e.g., covered vs. uncovered, height loss measurements) [55,56,57].

Previous reports on collagen irradiation suggest that it can alter the porosity, mechanical properties, and/or swelling behavior of collagen materials, indirectly supporting our observations of accelerated early absorption at 25–40 kGy; despite this, the previous studies were conducted under conditions differing from those of the current experiment (e.g., covered vs. uncovered, height loss measurements) [58].

Similarly, investigations of pore architecture and orientation highlight the influence of anisotropy on fluid transport, but they typically do not integrate occlusion and long-term hydration dynamics within a single experimental framework [59,60,61]. Our results address this gap, demonstrating that the rate—not merely the extent—of sponge hydration can be moderated by both application conditions (occlusion, pore orientation) and sterilization parameters (radiation dose). The current findings provide comprehensive data facilitating understanding of the factors governing hydration kinetics, offering a basis for optimizing collagen-based wound dressings for specific clinical applications.

## 4. Conclusions

Fish-originated collagen-based porous material was developed through the freeze-drying of collagen gel. The developed collagen sponge or scaffold is expected to be used as a wound dressing. The sponge comprises incorporated nanodiamonds, the presence of which does alter the properties of the material.

The manufacturing method imposes a specific bilayer microstructure of the spongy material with microporous (on the top) and microchannel (at the lower part of the material) structures. In either part, developed interconnected and heterogeneous porosity may be suitable for biomedical applications; in particular, it is favorable for fluid transport and potential cellular infiltration. As the mechanical property testing revealed maximum tensile stress at a moderate level of 45.9 kPa and relatively small elongation at break of less than 10%, this non-elastic material may require a plasticizer (introducing an additional substance, which may not be beneficial with regards to the safety and sterilizability of a medical device) or a supporting film or carrier fabrics in order to maintain the sponge integrity during application on a wound in the foreseen application. The results indicate that the incorporation of nanodiamonds does not affect the mechanical properties of the collagen material, but contrariwise irradiation with an electron beam does. Examination of the impact of ionizing radiation was our primary interest, as the radiation appears to be the only reliable, and possible to validate, method of collagen sponge sterilization on an industrial scale. The tensile stress of the porous material was reduced by more than 20% upon irradiation with doses of 25–40 kGy, a reasonable range for the sterilization of animal-derived biomaterials and products.

If applied as a wound dressing, collagen porous material can hydrate and absorb as demonstrated in the experiments with a hydrogel substrate mimicking a wound. It was a presence of a cover, simulating a dressed wound, that was the primary factor determining absorption kinetics. Occlusive conditions substantially accelerated both the hydration rate and the subsequent structural degradation of the sponges compared to uncovered conditions. Irradiation, used as a means of sterilization, at 25 kGy significantly accelerated early-stage hydration and loss of integrity in a moist environment, with a marginal further increase in the initial rate of hydration after increasing the dose to 40 kGy. As the fabricated porous materials have a bilayered structure, the orientation of the sponge relative to the hydrogel substrate (or a wound in actual application) also influenced hydration kinetics, demonstrating that structural anisotropy modulates fluid uptake depending on which surface interfaces with the hydrogel. Microchannels being in direct contact with the hydrogel accelerated the hydration and disintegration of the sponge.

The incorporation of nanodiamonds did not substantially affect the absorption kinetics of the collagen material, indicating that the primary determinants of fluid uptake are, besides the irradiation, the sponge internal architecture and orientation rather than its modification with nanoparticles. It is anticipated that surface-modified NDs to confer specific biological functionalities (e.g., antimicrobial activity) will also not alter the structural integrity or physicochemical properties of the collagen sponge. Consequently, the present results may be regarded as a reliable indication of the performance of hybrid or composite collagen-based porous structures and serve as support for the engineering of advanced bioactive biomaterials with tailored properties for diverse biomedical applications. Biological evaluation, including cytocompatibility tests with relevant skin cell lines (e.g., keratinocytes or fibroblasts), is planned as part of future studies aimed at further validation of the material for wound dressing applications.

## Figures and Tables

**Table 1:** Collagen sponge samples and their designations.

	Non-Irradiated	Irradiated	
		25 kGy	40 kGy
Collagen	Col-0	Col-25	Col-40
Collagen with original NDs	ColND-0	ColND-25	ColND-40
Collagen with thermally treated NDs	ColNDm-0	ColNDm-25	ColNDm-40

**Table 2:** Pore size characteristics including standard deviation (SD) for the mean Feret diameter (min. threshold = 0.150 mm).

Sample ID	Pore Count (No.)	Mean Feret ∅ [μm]
Col-0	124	285 ± 180
Col-0	118	310 ± 328
Col-0	115	265 ± 158
ColND-0	138	295 ± 177
ColNDm-0	145	305 ± 211

**Table 3:** Tensile properties of collagen sponge with and without irradiation.

		Deformation		Stress	
Material	Irradiation [kGy]	Average [%]	SD [%]	Average [kPa]	SD [kPa]
Col	0	6.1	1.3	53.3	9.5
	25	6.3	0.5	44.6	2.4
	40	6.2	1.1	40.5	7.9
ColND	0	8.0	1.6	52.3	11.1
	25	6.9	1.5	42.4	6.5
	40	8.3	1.1	45.4	5.9
ColNDm	0	6.9	1.5	51.7	8.4
	25	6.2	1.0	39.8	6.3
	40	6.1	2.1	42.6	7.4
	Average-0	7.0	1.5	52.4	9.7
	Average-25	6.5	1.0	42.3	5.1
	Average-40	6.9	1.4	42.8	7.1
	Average	6.8	1.3	45.8	7.3

**Table 4:** Time required for 50% sponge hydration, t_1/2_ [min] (microchannel vs. microsponge sides facing down).

Condition	Sample	Irradiation [kGy]	t_1/2_	SD t_1/2_
Covered	Microchannel side	0	89.3	6.7
		25	24.3	3.2
	Microsponge side	0	128.4	4.6
		25	54.1	7.4
Uncovered	Microchannels side	0	- *	- *
		25	300	7.1

* did not reach half of its heigh (t_1/2_) during the experiment time span of 450 min.

## Data Availability

The original data of the study are included in the article; further inquiries can be directed to the corresponding author.
